# Comparison of Characteristics of Neuropathic and Non-neuropathic Pruritus to Develop a Tool for the Diagnosis of Neuropathic Pruritus: The NP5

**DOI:** 10.3389/fmed.2019.00079

**Published:** 2019-04-17

**Authors:** Johanna Huguen, Emilie Brenaut, Caroline-Jade Clerc, Florence Poizeau, Pascale Marcorelles, Gaëlle Quereux, Alain Dupuy, Laurent Misery

**Affiliations:** ^1^Department of Dermatology, University Hospital, Brest, France; ^2^Department of Dermatology, Quimper Hospital, Quimper, France; ^3^Laboratoire Interactions Epitheliums Neurones, Université de Bretagne Occidentale, Brest, France; ^4^Department of Dermatology, University Hospital, Rennes, France; ^5^UPRES EA 7449 REPERES Pharmacoepidemiology and Health Services Research, Rennes University, Rennes, France; ^6^Department of Pathology, University Hospital, Brest, France; ^7^Department of Dermatology, University Hospital, Nantes, France

**Keywords:** neuropathic, pruritus, small fiber neuropathies, questionnaire, diagnosis

## Abstract

The diagnosis of neuropathic pruritus (NP) may be difficult. The aim of this study was to compare the characteristics of both neuropathic pruritus and non-neuropathic pruritus (NNP) in order to elaborate a tool to help the diagnosis of NP without clinical examination. One hundred and seven patients were included: Fifty three in the NP group and Fifty four in the NNP group. In multiple regression, presence of twinges, absence of burning, worsening with activity, no worsening with stress, and relief with cold ambient temperature were independent factors that were associated with NP. A score of two criteria out of five was optimal to discriminate NP from NNP with a sensitivity of 76% and a specificity of 77%. Alloknesis, hyperknesis, or the ice cube test were not included because their evaluation is based on clinical examination. Future high-powered studies are needed to confirm the results of the present study.

## Introduction

Neuropathic pruritus (NP) is related to neuronal or glial damages (1) at any point along the afferent sensory pathway of the nervous system (2). It is often underdiagnosed, although it affects ~8% of people with chronic pruritus, which is quite high for the general population (3). Both central nervous system (CNS) and peripheral nervous system (PNS) disorders can induce NP. Currently, the pathogenesis of NP requires further clarification (4).

The diagnosis of NP may be difficult. Dermatological and neurological examination associated with paraclinical examinations (CT scan, MRI, or skin biopsies) can be necessary to confirm the diagnosis (5). For example, MRI or CT-scan is recommended in brachioradial pruritus or notalgia paresthetica to identify root compression (3, 6).

Some sensations are reported to be occasionally associated with NP: burning, wet sensations, electric shocks, pins and needles, and pain (3, 7). Clinical examination may also determine alloknesis (i.e., an itchy or pruriceptive sensation evoked by a stimulus that is normally non-pruriceptive) or hyperkinesis (i.e., abnormal pruriceptive state in which a normally pruritic stimulus elicits a greater than normal duration and/or magnitude of itch) (2, 5). Alloknesis is quite similar to allodynia, which is a common phenomenon in neuropathic pain where a non-noxious stimulus can induce pain. The therapy is also challenging as the common antipruritic drugs are often ineffective for NP (antihistaminergic, topical steroids).

Our aim was to develop a screening questionnaire for NP, like previously made for neuropathic pain. Indeed, the development of a simple questionnaire based on verbal self-reporting of qualitative aspects of pain (as burning pain or electric shocks) has dramatically improved diagnosis and management of neuropathic pain in the past decade (8).

To our knowledge, there is no study comparing symptoms of neuropathic and non-neuropathic pruritus (NNP). The aim of this study was to compare the characteristics of both NP and NNP in order to develop a new NP diagnostic questionnaire. We were inspired by the methodology of Bouhassira et al. when they developed the Douleur Neuropathique 4 (DN4) questionnaire for the diagnosis of neuropathic pain (8).

## Patients and Methods

### Patients

Ethical review and approval was not required for this study in accordance with the national legislation and institutional requirements.

Adults with pruritus of at least moderate severity (mean or actual ≥3 out of 10 on a numeric scale) were included in the study. Inclusion criteria in the NP group was a diagnosis of NP among small fiber neuropathy (SFN), polyneuropathies, notalgia paresthetica, post-radiation therapy, brachioradial pruritus, or posterior cervical myelopathy. Diagnosis was based on clinical examination, and confirmed by paraclinical examinations when necessary: skin biopsies showing a reduced intraepidermal nerve fiber density measurements in the distal leg for SFN, CT scan, or MRI to look for the etiology of notalgia paresthetica or brachioradial pruritus, electroneuromyography for polyneuropathies. In the NNP group, inclusion criteria was a diagnosis of itchy dermatitis (as psoriasis, eczema) or a psychogenic pruritus. Exclusion criteria were as follows: patients under 18 years old, the inability to complete the questionnaire because of cognitive or physical impairment, and pruritus of unknown or mixed origin.

A patient with NNP was recruited for each included patient with NP. The patients were recruited during hospitalization or consultation in the Dermatology department of four centers: Brest, Rennes, Nantes, and Quimper Hospitals, with questionnaires directly given to them.

This study was carried out with the written consent of all patients.

### Development of the Questionnaire

On the basis of clinical experience and analysis of the literature, we reported a list of signs and symptoms that could be associated with NP to construct this survey (2–4, 7, 9). The questionnaire derived from this list included questions in order to determine the history of symptoms, characteristics of pruritus, frequency, localization, intensity, and modulating factors of pruritus.

Concerning the characteristics of pruritus, the presence of the following sensory descriptors was evaluated: pain, pins and needles, tingling, twinges, pinching, burning, electric shocks, numbness, and painful cold.

Questions were also asked about different worsening and alleviating factors. This questionnaire was not intended to be exhaustive and was deliberately restricted to a minimum of simple questions. The survey was based on patient interview only and was 2 pages long. The estimated time to fill out the questionnaire was around 5 min.

### Statistical Analysis

All data were analyzed using RStudio Version 1.0.136 (RStudio, Inc, Boston, MA, USA). Our study was analyzed in descriptive and analytic methods. The quantitative variables were described using mean and standard deviation. Means were compared with Student's *T*-test. The qualitative variables were described using frequency and percentages. For each item, the proportion of positive responses was compared between the two populations: NP and NNP using a Chi2 test or Exact Fisher's test in case of small sample sizes. For all statistical analyses the type 1 error has been set at 5%. Sensory descriptors and pruritus modifying factors that correlated with the neuropathic pruritus (NP) were evaluated using a univariate logistic regression analysis first. To eliminate potential confounders, each item was tested for independence by the multivariate forward stepwise regression analysis. To evaluate the predictive efficiency of a questionnaire for the diagnosis of NP based on clinical independent predictive factors, receiver operating characteristic (ROC) curve was drawn and sensitivity/specificity were calculated.

## Results

Patients were included between March 2016 and November 2017. In the NP group, 99 questionnaires were sent, and 59 patients answered (response rate 59.6%). Four patients were excluded because of low pruritus intensity, and two declined to participate. In the NNP group, 35 questionnaires were given during consultation or hospitalization (15 in Brest, 8 in Rennes, 5 in Nantes, and 7 in Quimper). Forty-four questionnaires were sent to patients meeting the inclusion criteria who were hospitalized for dermatological pruritus during the same period in the department of dermatology in Brest University hospital. Twenty-one patients responded (response rate 47.7%), and two patients were excluded because of low pruritus intensity. A total of 107 patients were included: 53 in the NP group and 54 in the NNP group. Demographic characteristics and etiologies of pruritus are presented in Supplementary Table 1. Characteristics of pruritus in NP and NNP are presented in Table 1.

**Table 1:** Characteristics of pruritus in neuropathic and non-neuropathic pruritus.

	**Neuropathic pruritus *n* = 53**	**Non-neuropathic pruritus *n* = 54**	***p*-value**
**Intensity (Mean** **±** **SD)**			
Maximum	8.3 ± 1.9	8.5 ± 1.7	0.606
Minimum	2.4 ± 2.3	2.1 ± 2.2	0.451
Mean	5.5 ± 1.9	5.3 ± 1.7	0.640
**Frequency** ***n*** **(%)**			
Everyday	44 (83)	40 (74)	0.581
Almost daily	7 (13)	9 (17)	
Weekly	2 (4)	2 (4)	
Monthly	0 (0)	2 (4)	
Rarely	0 (0)	1 (2)	
**Duration** ***n*** **(%)**			
Weeks^*^	1 (2)	4 (7)	0.006
Months	8 (15)	20 (37)	
Years	44 (83)	30 (56)	
**Rhythm** ***n*** **(%)**			
Continuous	19 (36)	18 (33)	0.784
Intermittent	34 (64)	36 (67)	
**Scratching** ***n*** **(%)**			
Never^*^	3 (6)	0 (0)	0.002
Rarely	10 (19)	2 (4)	
Often	15 (28)	30 (56)	
Very often	25 (47)	22 (41)	
**Pleasurability of Scratching** ***n*** **(%)**			
Very pleasant	10 (19)	9 (17)	0.890
Moderately pleasant	17 (32)	21 (39)	
Neutral	4 (8)	4 (7)	
Moderately unpleasant	9 (17)	6 (11)	
Very unpleasant	13 (24)	14 (26)	

* *p < 0.05*.

### Univariate Analysis

Concerning sensory descriptors, twinges (*p* = 0.030) and electric shocks (*p* = 0.024) were significantly associated with NP (Supplementary Figure 1). Concerning pruritus modifying factors, a list of factors potentially impacting pruritus was proposed (worsen, does not affect, or alleviate pruritus). The comparison of the two groups is reported in Table 2. Activity significantly worsened NP whereas stress significantly worsened NNP. Concerning alleviating factors, a comparison of the two groups is reported Table 3: cold ambient temperature significantly relieved NP.

**Table 2:** Pruritus worsening factors in neuropathic and non-neuropathic pruritus.

	**Neuropathic pruritus *n* (%)**	**Non-neuropathic pruritus *n* (%)**	***p*-value**
Sleep	21 (40)	23 (43)	0.755
Rest	15 (28)	19 (35)	0.445
Activity^*^	16 (30)	5 (9)	0.006
Stress^*^	25 (47)	37 (69)	0.025
Fatigue	20 (38)	27 (50)	0.201
Physical activity	17 (32)	14 (26)	0.483
Dry skin	30 (57)	39 (72)	0.091
Hot water	23 (43)	20 (37)	0.502
Cold water	4 (8)	6 (11)	0.742
Sweating	22 (42)	28 (52)	0.284
Cold ambient temperature	5 (9)	4 (7)	0.742
Hot ambient temperature	26 (49)	28 (52)	0.772
Some clothes	31 (58)	25 (46)	0.207
Friction	32 (60)	34 (63)	0.783

* *p < 0.05*.

**Table 3:** Pruritus alleviating factors in neuropathic and non-neuropathic pruritus.

	**Neuropathic pruritus *n* (%)**	**Non-neuropathic pruritus *n* (%)**	***p*-value**
Sleep	12 (23)	14 (26)	0.692
Rest	11 (21)	16 (30)	0.291
Activity	12 (23)	16 (30)	0.411
Stress	0 (0)	0 (0)	1
Fatigue	0 (0)	1 (2)	1
Physical activity	5 (9)	5 (9)	1
Dry skin	0 (0)	2 (4)	0.495
Hot water	7 (13)	8 (15)	0.811
Cold water	25 (47)	23 (43)	0.634
Sweating	1 (2)	0 (0)	0.495
Cold ambient temperature^*^	20 (38)	8 (15)	0.007
Hot ambient temperature	3 (6)	2 (4)	0.678
Some clothes	3 (6)	1 (2)	0.363
Friction	4 (8)	2 (4)	0.437

* *p < 0.05*.

### Multivariate Analysis

Multiple regression analysis of sensory descriptors and pruritus modifying factors showed that presence of twinges (*p* < 0.01), absence of burning (*p* = 0.03), worsening with activity (*p* < 0.01), no worsening with stress (*p* = 0.001), and relief with cold ambient temperature (*p* = 0.02) were independent factors that were associated with NP (Table 4).

**Table 4:** Correlation between clinical factors and neuropathic pruritus.

**Covariates**	**Odds-ratio**	**95% confidence interval**	***p*-value**
Twinges^*^	4.94	[1.70; 15.85]	< 0.01
Burning^*^	0.31	[0.10; 0.84]	0.03
Worsening with activity^*^	7.87	[2.21; 33.16]	< 0.01
Worsening with stress^*^	0.20	[0.07; 0.52]	0.001
Relief with cold ambient temperature^*^	3.62	[1.23; 11.57]	0.02

* *p < 0.05*.

### Five Clinical Independent Predictive Factors

Five criteria were significantly more present in NP than in NNP: presence of twinges, absence of burning, worsening with activity, no worsening with stress, relief with cold ambient temperature.

### Specificity and Sensitivity

A score of two criteria out of five was optimal to discriminate NP from NNP with a sensitivity of 76% and a specificity of 77%. Nobody had five criteria. The multivariate model achieved an area under the curve of 0.80. The ROC curve can be seen in Supplementary Figure 2.

## Discussion

Our study is the first to compare the clinical characteristics of NP and NNP. The aim of this study was to create a diagnostic questionnaire similar to the DN4 (8), which was obtained by comparing symptoms in patients suffering from pain of neuropathic or non-neuropathic origin. This 10-item questionnaire is based on the patient interview and clinical examination. A score of 1 is given to each positive item. A total score equal or more than 4 is necessary for the diagnosis of neuropathic pain. A simplified version (called DN4-i) can be used based on patient interview alone. The cut-off value for the diagnosis of neuropathic pain is 3 in this simplified version. DN4-i is a helpful tool to identify neuropathic pain in clinical research and daily practice. Using a similar methodology, we propose a new tool for the diagnosis of NP, that we called Neuropathic Pruritus 5 (NP5), based on significant differences (*p* < 0.05) between NP and NNP. The 5 questions (1 point if positive response) will be:

- is your pruritus associated with twinges,
- is your pruritus associated with burning,
- is your pruritus worse with activity,
- is your pruritus worse with stress
- is your pruritus relieved by cold ambient temperature.

In the literature, NP was shown to be accompanied with pain, allodynia, paresthesia, hypo- or hyper-esthesia, electric shocks and twinges (3, 4, 10), which is congruent with our results. Twinges were particularly reported in herpes-zoster-related itch and pain (10). Pruritus is mediated by low-conducting unmyelinated C-fibers (mechano- and heat-insensitive), which are functionally distinct from C-fibers involved in pain mediation (5, 11). Primary afferent Aδ fibers are also involved in pruritus mediation, particularly in the alloknesis and hyperkinesis phenomenon due to access to pruritoceptive dorsal horn neurons. Complex interactions exist between pain and itch conduction at the spinal level (11). NP is supposedly accompanied by neuropathic pain (4, 12), which refers to all pains initiated or caused by a primary lesion or dysfunction of the nervous system, according to the definition of the International Association for Study of Pain (IASP).

Surprisingly, we did not find that pain was significantly more frequent in the NP group. This is probably because pain is also frequently reported in NNP, such as eczema, atopic dermatitis, psoriasis, scabies, and urticarial (13). Another recent study emphasized that skin pain is a common and burdensome symptom in atopic dermatitis (14). Hence, pain cannot be used to discriminate pruritus associated with nerve injury from NNP. Similarly, pins and needles, and relief with cold water were also similar in the two groups. In the literature, these symptoms and modifying factors were described with NP (3, 7, 15). On the other hand, a French study also emphasized that stingings (i.e., pins and needles) as well as alleviating with cold water were frequent symptoms in dermatological pruritus (7). Consequently, they are not specific for NP.

Moreover, numbness could be a specific criterion and tends to be effective in NP in our study, but no significant difference was found, which may be due to low statistical power. We should also note that other triggering factors were similar between the two groups: sweating, friction, wearing some clothes, hot water and hot ambient temperature are common in pruritus of various origins (7, 13, 15, 16). Moreover, pruritus intensity and frequency were similar in the two groups. Pleasure from scratching was similar in NP and NNP as well.

We also found that three modulating factors were significantly different in the two groups: NP was relieved with cold ambient temperature, increased with activity but not worsened with stress. Hoitsma et al. also reported that activity was a worsening factor in SFN (17). This purpose was not specific for itch but all symptoms of SFN. Bernhard et al. highlighted that brachioradial pruritus could be relieved with cold application as in the pathognomonic “ice-packing sign,” although cold ambient temperature was not reported (6).

Stress is a common worsening factor in dermatological pruritus in the literature (18). Surprisingly, this modulating factor was significantly different between the two groups as stress significantly worsened NNP. It may be related to a high number of patients with psychogenic pruritus in our sample (20% of NNP). In the literature, Dalgard et al. highlighted that there was a strong association between itch and psychosocial factors (18). In this Norwegian study, individuals reporting itch were more distressed, experienced more negative life events and had poorer social support. Another Norwegian study showed the same results with adolescents (19).

Some study limitations should be considered. We did not choose to evaluate alloknesis, hyperknesis, or the ice cube test because clinical evaluation would have been necessary, although the study was deliberately based on an interview without clinical examination. Further studies could add clinical examination to the patient interview to increase information gathered on the five interview-specific symptoms in order to lead to a diagnosis score for NP in routine practice.

Usually, the diagnosis of neuropathic itch is based on the presence of clinical signs, abnormal sensations and neurological symptoms and/or localization along dermatomes, and can often be straightforward. In a previous paper (4), we suggested that “after careful dermatological and neurological examination, paraclinical examinations should be conducted to confirm the diagnosis and the etiology of the condition. The suggested tests include skin biopsies to measure IENF density, as well as electromyography, sural nerve conduction studies, QST, and MRI.” We used this methodology to get diagnosis. To our knowledge, there are no consensual diagnostic criteria for NP. We choose to include a heterogenous group of non-neuropathic patients to allow comparisons with neuropathic patients, as previously done for the study on neuropathic pain (8).

Despite statistical differences between the two groups (NP and NNP), the clinical relevance of some of the shown differences should be interpreted with caution. Especially regarding the absence of burning, which despite statistical significant difference between NP and NNP, the percentage of affected patients seems almost similar in both groups. To conclude, the five questions of our tool Neuropathic Pruritus 5 (NP5) can be asked to help diagnose NP. Two or more positive criteria are in favor of NP with a sensitivity of 76% and specificity of 77%. Further validation studies are needed for the external validation of the questionnaire, as Bouhassira et al. did for the development of the neuropathic pain questionnaire (20). The choice of the 5 questions for the diagnosis of NP would be confirmed (or not). Moreover, other studies comparing patients with neuropathic pruritus and patients with pruritus related to other conditions (renal, hepatic hematological, or others) would be very interesting.

## Ethics Statement

Ethical review and approval was not required for this study in accordance with the national legislation and institutional requirements.

## Author Contributions

JH, EB, and LM wrote the manuscript. EB and LM designed the work. All authors performed acquisition, analysis or interpretation of data for the data, revised the manuscript and provided approval for publication of the content.

### Conflict of Interest Statement

The authors declare that the research was conducted in the absence of any commercial or financial relationships that could be construed as a potential conflict of interest.

## References

[1] Ständer S Weisshaar E Mettang T Szepietowski JC Carstens E Ikoma A. Clinical classification of itch: a position paper of the International Forum for the Study of Itch. Acta Derm Venereol. (2007) 87:291–4. 10.2340/00015555-0305 17598029

[2] Yosipovitch G Samuel LS. Neuropathic and psychogenic itch. Dermatol Ther. (2008) 21:32–41. 10.1111/j.1529-8019.2008.00167.x 18318883

[3] Stumpf A Ständer S. Neuropathic itch: diagnosis and management. Dermatol Ther. (2013) 26:104–9. 10.1111/dth.12028 23551367

[4] Misery L Brenaut E Le Garrec R Abasq C Genestet S Marcorelles P. Neuropathic pruritus. Nat Rev Neurol. (2014) 10:408–16. 10.1038/nrneurol.2014.99 24912513

[5] Binder A Koroschetz J Baron R. Disease mechanisms in neuropathic itch. Nat Clin Pract Neurol. (2008) 4:329–37. 10.1038/ncpneuro0806 18461071

[6] Bernhard JD Bordeaux JS. Medical pearl: the ice-pack sign in brachioradial pruritus. J Am Acad Dermatol. (2005) 52:1073. 10.1016/j.jaad.2005.02.056 15928630

[7] Brenaut E Marcorelles P Genestet S Ménard D Misery L. Pruritus: an underrecognized symptom of small-fiber neuropathies. J Am Acad Dermatol. (2015) 72:328–32. 10.1016/j.jaad.2014.10.034 25484269

[8] Bouhassira D Attal N Alchaar H Boureau F Brochet B Bruxelle J. Comparison of pain syndromes associated with nervous or somatic lesions and development of a new neuropathic pain diagnostic questionnaire (DN4). Pain. (2005) 114:29–36. 10.1016/j.pain.2004.12.010 15733628

[9] Oaklander AL. Common neuropathic itch syndromes. Acta Derm Venereol. (2012) 92:118–25. 10.2340/00015555-1318 22307048

[10] Elkersh MA Simopoulos TT Malik AB Cho EH Bajwa ZH. Epidural clonidine relieves intractable neuropathic itch associated with herpes zoster-related pain. Reg Anesth Pain Med. (2003) 28:344–6. 10.1016/S1098-7339(03)00182-2 12945030

[11] Ikoma A Steinhoff M Ständer S Yosipovitch G Schmelz M. The neurobiology of itch. Nat Rev Neurosci. (2006) 7:535–47. 10.1038/nrn1950 16791143

[12] Yosipovitch G Carstens E McGlone F. Chronic itch and chronic pain: analogous mechanisms. Pain. (2007) 131:4–7. 10.1016/j.pain.2007.04.017 17524558

[13] Brenaut E Garlantezec R Talour K Misery L. Itch characteristics in five dermatoses: non-atopic eczema, atopic dermatitis, urticaria, psoriasis and scabies. Acta Derm Venereol. (2013) 93:573–4. 10.2340/00015555-1599 23722181

[14] Vakharia PP Chopra R Sacotte R Patel KR Singam V Patel N. Burden of skin pain in atopic dermatitis. Ann Allergy Asthma Immunol. (2017) 119:548–52. 10.1016/j.anai.2017.09.076 29223299 PMC5726579

[15] Pereira MP Lüling H Dieckhöfer A Steinke S Zeidler C Ständer S. Brachioradial pruritus and notalgia paraesthetica: a Comparative Observational Study of clinical presentation and morphological pathologies. Acta Derm Venereol. (2017) 98:82–8. 10.2340/00015555-2789 28902951

[16] Murota H Katayama I. Exacerbating factors of itch in atopic dermatitis. Allergol Int. (2017) 66:8–13. 10.1016/j.alit.2016.10.005 27863904

[17] Hoitsma E Reulen JP de Baets M Drent M Spaans F Faber CG. Small fiber neuropathy: a common and important clinical disorder. J Neurol Sci. (2004) 227:119–30. 10.1016/j.jns.2004.08.012 15546602

[18] Dalgard F Lien L Dalen I. Itch in the community: associations with psychosocial factors among adults. J Eur Acad Dermatol Venereol. (2007) 21:1215–19. 10.1111/j.1468-3083.2007.02234.x 17894708

[19] Halvorsen JA Dalgard F Thoresen M Thoresen M Bjertness E Lien L. Itch and mental distress: a cross-sectional study among late adolescents. Acta Derm Venereol. (2009) 89:39–44. 10.2340/00015555-0554 19197540

[20] Bouhassira D Lantéri-Minet M Attal N Laurent B Touboul C. Prevalence of chronic pain with neuropathic characteristics in the general population. Pain. (2008) 136:380–7. 10.1016/j.pain.2007.08.013 17888574

