# Supplementary material for: Comparison of Characteristics of Neuropathic and Non-neuropathic Pruritus to Develop a Tool for the Diagnosis of Neuropathic Pruritus: The NP5
Source: Front Med (Lausanne). 2019 Apr 17;6:79. doi: 10.3389/fmed.2019.00079 (PMC6499201; doi:10.3389/fmed.2019.00079)
Supplement: Supplementary file 1 [file Table_1.docx]

|  | **Neuropathic pruritus**  **n=53** | **Non-neuropathic pruritus**  **n=54** | **p value** |
| --- | --- | --- | --- |
| ***Sex*** *n (%)*  *Male  Female* | 22 (42)  31 (58) | 33 (61)  21(39) | 0.042 |
| **Age** *(years)*  *Mean +/- SD* | 66.5 +/- 12.8 | 61.9 +/- 18.8 | 0.143 |
| **Etiologies**  n (%) | \| SFN \| 45(85) \| \| --- \| --- \| \| Brachioradial pruritus \| 3(6) \| \| Post-radiation therapy \| 1(2) \| \| Notalgia paresthetica \| 2(4) \| \| Axonal polyneuropathy \| 1(2) \| \| Posterior cervical myelopathy \| 1(2) \| | \| Psoriasis \| 13(24) \| \| --- \| --- \| \| Eczema \| 13(24) \| \| Atopic dermatitis \| 1(2) \| \| Psychogenic pruritus \| 11 (20) \| \| Urticaria \| 2(4) \| \| Scabies \| 1(2) \| \| Bullous pemphigoid \| 5(9) \| \| Mycosis fungoid \| 3(6) \| \| Sezary syndrome \| 4(7) \| \| Pityriasis rubra pilaris \| 1(2) \| |  |

Supplementary table 1. Demographic characteristics and etiologies of pruritus in the two groups: neuropathic and non-neuropathic pruritus.
