# Supplementary figures and images for: Comparison of Characteristics of Neuropathic and Non-neuropathic Pruritus to Develop a Tool for the Diagnosis of Neuropathic Pruritus: The NP5
Source: Front Med (Lausanne). 2019 Apr 17;6:79. doi: 10.3389/fmed.2019.00079 (PMC6499201; doi:10.3389/fmed.2019.00079)

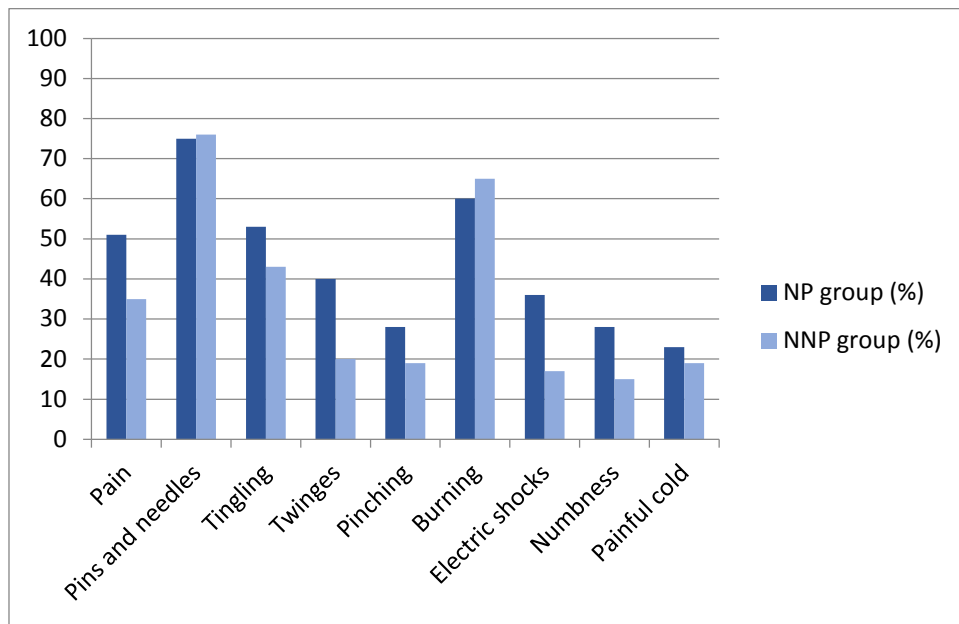

Figure 1. Frequency of sensory descriptors in NP and NNP

Supplement: Supplementary file 2 [file Image_1.pdf]

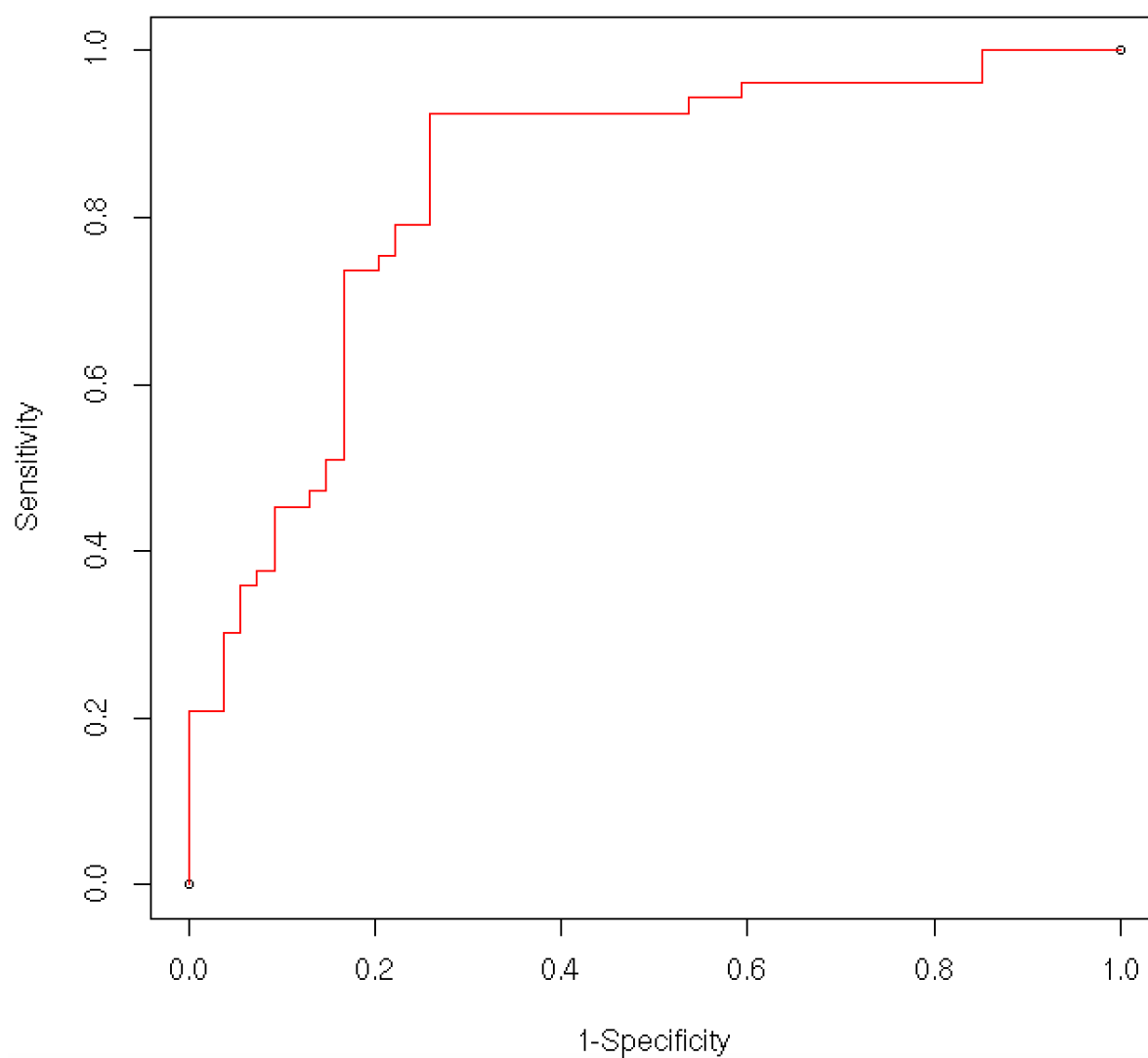

Figure 2: ROC Curve of multivariate model to discriminate NP from NNP

Supplement: Supplementary file 3 [file Image_2.pdf]
